# A Systematic Method for Exploring Data Attributes in Preparation for Designing Tailored Infographics of Patient Reported Outcomes

**DOI:** 10.5334/egems.190

**Published:** 2018-01-24

**Authors:** Adriana Arcia, Janet Woollen, Suzanne Bakken

**Affiliations:** 1 Columbia University, US

**Keywords:** Patient Centered Care, Information Visualization, Infographics, Patient Reported Outcomes, Data Attributes, Precision Medicine

## Abstract

**Context:**

Tailored visualizations of patient reported outcomes (PROs) are valuable health communication tools to support shared decision making, health self-management, and engagement with research participants, such as cohorts in the NIH Precision Medicine Initiative. The automation of visualizations presents some unique design challenges. Efficient design processes depend upon gaining a thorough understanding of the data prior to prototyping.

**Case Description:**

We present a systematic method to exploring data attributes, with a specific focus on application to self-reported health data. The method entails a) determining the meaning of the variable to be visualized, b) identifying the possible and likely values, and c) understanding how values are interpreted.

**Findings:**

We present two case studies to illustrate how this method affected our design decisions particularly with respect to outlier and non-missing zero values.

**Major Themes:**

The use of a systematic method made our process of exploring data attributes easily manageable. The limitations of the data can narrow design options but can also prompt creative solutions and innovative design opportunities.

**Conclusion:**

A systematic method of exploration of data contributes to an efficient design process, uncovers design opportunities, and alerts the designer to design challenges.

## Context

Tailored visualizations of health information such as patient reported outcomes (PROs) can be used to enhance health communication, particularly to patients and community members with limited health literacy [1]. Visualizations can serve multiple communication purposes including engaging the viewer, supporting comprehension, motivating behavior change, delivering a persuasive message, and much more [2, 3]. Visualization as a form of health communication – in the context of the availability of large amounts of health data – is increasingly important for several reasons. One, the expectation that clinical encounters will involve shared decision making necessitates informational tools to support the process. Two, individuals need tools to understand and track their health data to engage in effective health self-management. Three, researchers need tools to engage with participants and the larger community around research results. Such engagement is more important now than ever as we move into the era of precision medicine which has been broadly defined by the National Institutes of Health (NIH) as an emerging approach for disease treatment and prevention that takes into account individual variability in genes, environment, and lifestyle for each person. The NIH Precision Medicine Initiative (PMI) has recruitment of a diverse cohort as a goal and includes plans for communicating research results as a mechanism of engagement [4].

In order to be individualized for the viewer with her/his own health data, the generation of tailored visualizations must be automated in order to be scalable. Compared to single-use visualizations, automated visualizations present additional, unique design challenges when the objective is a consistently effective output. Numerous tools are available for matching data types to chart types and for automating visualizations (see Table 1), but often these are general purpose – not health focused – and most require the user to possess both domain knowledge and visualization design expertise [5]. Prominent existing health visualizations have risk communication as a focus, [6, 7]. leaving many other aspects of health communication unexplored. We perceived a lack of resources for visualizing personal health data with patients and community members as the intended audience. Therefore, the focus of our work has been on creating culturally relevant, automatically tailored visualizations of PROs specifically designed to meet the needs of community members with varying levels of health literacy. As ours and others’ efforts advance, we may ultimately be able to contribute to a bank of visualizations for PROs, particularly those from the Patient Reported Outcomes Measurement Information System (PROMIS) [8], or for a variety of other self-reported and patient-generated data.

**Table 1:** Selected Visualization Resources.

Title and URL	Description

Visualizing Health *http://www.vizhealth.org/*	Evidence based risk-communication visualizations
Icon Array Generator *http://www.iconarray.com/*	Icon arrays display part-to-whole relationships for communicating health risks
Data Viz Project *http://datavizproject.com/*	Interactive directory of visualization formats categorized by family, function, shape, and input
Chart Chooser *http://labs.juiceanalytics.com/chartchooser/index.html*	Use filters to find the right visualization for the data and download as Excel or PowerPoint templates
Choosing A Good Chart *http://extremepresentation.typepad.com/blog/2006/09/choosing_a_good.html*	Decision tree for chart selection
Graphic Cheat Sheet *http://billiondollargraphics.com/graphic-cheat-sheet/*	Interactive chart selection tool
Properties And Best Uses Of Visual Encodings *http://complexdiagrams.com/properties*	Suggested encoding elements according to data characteristics
See *http://selection.datavisualization.ch/* and *http://dataviz.tools/* for an extensive curated selection of data visualization tools.	

Our visualization work began in the context of the Washington Heights/Inwood Informatics Infrastructure for Community-Centered Comparative Effectiveness Research (WICER) project. For WICER, numerous PROs, including some PROMIS measures, were collected from over 5,800 members of the Washington Heights/Inwood community in Northern Manhattan. In light of the varying levels of health literacy present in the community, we elected to develop information visualizations (infographics) to communicate survey data back to the respondents who so generously contributed it. Our goal was to provide the information in a culturally relevant, easily comprehensible format that respondents could use to support their health self-management efforts. Briefly, the process we employed began with initial prototyping by a multi-disciplinary working group [9], followed by iterative prototyping with community feedback [10]. In order to automate the tailoring of the resulting infographic designs, we developed a system called EnTICE:3 **E**lectro**n**ic **T**ailored **I**nfographics for **C**ommunity **E**ngagement, **E**ducation, and **E**mpowerment [11]. The last step is formal comprehension testing, one approach to which is an experimental protocol that evaluates the extent the infographics support comprehension compared to text alone [12].

Having developed a start-to-finish method for designing visualization of PROs, we are now applying the method to new PROs from the New York City Hispanic dementia caregiver Research Program (NHiRP). For NHiRP, the purpose of the visualizations will be to support Hispanic caregivers’ health self-management and caregiving efforts. Revisiting the early steps of the design process has served to highlight the utility of using a systematic method to get to know the data to be visualized. We know from experience that it is not sufficient to just broadly identify the data type (e.g., categorical, continuous) and move forward. A deeper dive is necessary for a streamlined design process. After all, “if a poor choice was made in the abstraction stage, then even perfect visual encoding and algorithm design will not create a visualization system that solves the intended problem” (p. 921) [13]. The purpose of this paper is to share our systematic method to understanding the data. It represents a synthesis of best practices and key lessons from experience. Our focus has been on PROs and other self-reported data, but the proposed method may have relevance to numerous data types, such as the various types of data collected in clinical encounters. The considerations we present may be familiar to those working in information visualization and increasingly, journalism. Our goal is to present these data considerations from a biomedical informatics perspective with a specific focus on application to health data. As such, our method may be of greatest interest to data analysts embarking on health care projects and other health care professionals who will design visualizations.

### Relevant Literature

Some recent literature from the Information Visualization (InfoVis) community is relevant to this topic. Huang and colleagues have contributed a taxonomy of the data, contexts, interactions, and insights that describe personal visualization and personal visual analytics [14]. Meyer and colleagues proposed the Nested Blocks and Guidelines Model intended to guide the design and validation of visualization systems [13, 15]. From high to low, the levels are domain characterization, data/task abstraction design, encoding interaction/technique design, and algorithm design; the model specifies the nature of within-level and between-level guidelines. The work presented in this paper falls into the level of data/task abstraction design. In the process of getting to know the data, readers may appreciate how Bigelow et al., conceptualize data attributes [16]. Data semantics relates to the meaning of the data. Data behavior describes the shape of the data and the patterns found therein. Data structure (aka data type) reflects decisions about the organization of the data. Derived data are the result of transformations and manipulation of the original data. Data structure and derived data are known collectively as data abstraction; that is, the “specific interpretation of data that supports the high-level goals of visualization” (p. 18) [16]. The systematic method presented in this paper concerns data abstraction. Within the medical informatics literature, Kosara & Miksch reviewed methods for visualization of measurements, display of incidents, and depiction of planned actions [17], however, the target audience for the visualization described in their review is clinicians rather than patients and community members.

## Case Description

In this paper we assume that the visualization designer has already engaged in the critical step of clearly elucidating the goals and desired outcomes of visualization [9]. With the purpose of the visualization identified, we propose getting to know the attributes of the data following our systematic method of a) determining the meaning of the variable, b) identifying the possible and likely values, and c) understanding how values are interpreted. We present two case studies to illustrate how this method affected our design decisions.

### What Does the Variable Mean?

Our first step in getting to know our data in advance of visualization was gaining an understanding of precisely what had been measured. If the variable is observed, such as cans of soda consumed per week, the meaning is typically straightforward. If the variable is latent, such as depression, the specific meaning may be more nuanced. For example, depending upon how it is scored, the PHQ-9 (a measure of depression) can provide information about whether criteria have been met for a diagnosis of depression or it may inform about symptom severity irrespective of diagnosis [18]. Different visualization opportunities may arise depending upon subtleties of meaning; in the former case the focus may be on the diagnostic threshold whereas in the latter the visual message is likely to be about intensity/degree.

Latent variables are sometimes collected using a single item such as item A in Table 2 (known as SF1) [19]. More commonly, though, an instrument comprising multiple items is used. In either case, it is advisable to start by identifying the underlying construct.

**Table 2:** Item examples referenced in the text.

Item Stem	Response Options	Comments

A. “In general, would you say your health is…?” [19]	Poor, Fair, Good, Very good, Excellent	Unipolar response options; increasing health
B. “Compared with 10 years ago, how is [care recipient] at recognizing the faces of family and friends?” [21]	Much improved, A bit improved, Not much change, A bit worse, Much worse	Bipolar response options with neutral midpoint; change in signs of dementia
C. “I can make time for physical activity.” [22]	Strongly agree, Agree, Disagree, Strongly disagree	Bipolar response options with no midpoint; supports to physical activity
D. Systolic blood pressure	mmHg in whole numbers	General population cutpoints at 120 and 140 mmHg [23]

- *Do the authors of the instrument identify a theoretical framework that guided instrument development? Do they provide a clear definition of the underlying construct?* In cases where the theoretical underpinnings are not explicitly stated, they must be inferred from the content of the items (face validity).
- *What is the meaning of the items? How are respondents and/or viewers of the visualization likely to interpret them?* Variables that, at first glance, appear to be straightforward, may not be when respondent interpretation is taken into account. For example, a substantial proportion of the general public will have a culturally-based understanding of what constitutes a “serving” of vegetables that may or may not align with current dietary guidelines. Therefore, a graphical depiction of measuring cups might be appropriate for participants in an organized weight reduction program but not appropriate for the general public.
- *What type of response is sought?* Common response types include frequency, intensity, duration, and level of agreement.

The answers to the above questions can provide fertile source material for identifying meaningful imagery for visualizations. One instrument used in NHiRP is the Kessler Psychological Distress Scale [20]. As a non-specific measure of psychological distress, the items in the Kessler tap multiple domains including depressed mood, anxiety, worthless guilt, fatigue, and motor agitation/retardation. These domains evoke imagery both individually and collectively that can be applied to visualization. Image searches for “distress” and “psychological distress” in Google and in clip art databases, resulted in the emergence of some consistent motifs. Chief among these were a) a slumped figure, head in hands; and b) rain, clouds, and/or lightning in or around a figure’s head. This kind of image search is useful for identifying the visual metaphors and symbolic analogies associated with the shared cultural understanding of a term. Of the designs developed for WICER, the ones that employed familiar color and/or symbolic analogies (e.g., a battery to represent sleep and energy) were often the most popular and readily understood [10].

### What Values Are Possible?

Our next step toward understanding the data was to identify the values that are possible for a given variable. Getting a sense of the range of typical values also was useful. Different considerations come into play for categorical/ordinal and continuous variables.

#### Categorical/Ordinal Variables

Variables that are categorical or ordinal typically have relatively few possible values and as such, are easier to plan for and visualize in a predictable way. To understand these variables, we considered:

- *What are the response options and how are they encoded?* The response options to item A in Table 2 might typically be coded as poor = 0, fair = 1, good = 2, very good = 3, excellent = 4. Being aware of the coding was necessary for understanding the typical values present within a population when the information was reported as a mean and standard deviation instead of a distribution. If one or more response options are never or very rarely used, it may be possible to omit them from consideration in visualization design.
- *Are responses unipolar or bipolar?* Unipolar response options, such as those in item A, suggest increasing quantities of the variable (e.g., “health”). Bipolar response options, as in item B, can be conceptualized as positive and negative values on a number line centered over zero.
- *Do bipolar response options include a neutral midpoint?* Bipolar response options imply a midpoint, but that midpoint is not always made available to respondents as in item C in Table 2.
- *What transformations, such as collapsing categories, are possible and/or desirable?* In the case of item B, the five response options could conceivably be collapsed down to three: improvement, no change, and worsening. For item C, responses could be dichotomized into agreement and disagreement.

#### Continuous Variables

**Figure 1:**
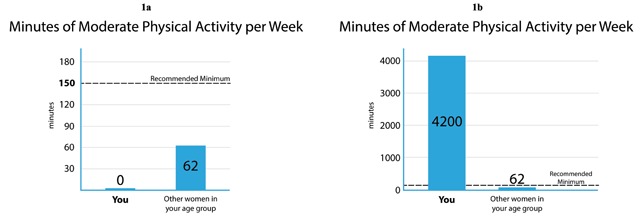
Infographics from WICER demonstrating design techniques to accommodate the effects of extreme values. In **(1a)** A very shallow bar is used to indicate where the bar would be if the value were not zero. The remaining visual elements are proportioned as the designer intended. In **(1b)** The need to accommodate a very high value obscures the recommended minimum value.

Some considerations we found useful for understanding continuous variables included:

- *What is the scaling or metric?* Celsius, *z*-scores, T-scores, meters, millimeters of mercury (mmHg), grams, beats per minute, minutes of physical activity, and servings of vegetables are all examples of metrics for observed continuous variables. Instruments that measure latent variables generally result in a total summed or averaged score that is treated as continuous despite the fact that responses were captured at the item level in a categorical or ordinal fashion. In this case, possible values depend upon the scoring: a hypothetical 10-item instrument with 5 response options per item could just as easily be scaled 0–40 as 10–50.
- *If zero is a possible value, what is its meaning?* For ratio measures such as meters, beats per minute, servings of vegetables, etc., zero signifies the absence of the phenomenon being measured. By contrast, zero for a *z*-score is benchmarked to the population mean.
- *What are the minimum and maximum possible values? What are the minimum and maximum observed values?* Metrics such as percentages have clear maximum values but often there is no theoretical maximum (e.g., meters, grams, minutes, etc.). However, even in cases where data collection has not occurred or is not complete, it is possible to make educated guesses about the limits of extreme values for familiar phenomena such as systolic blood pressure (item D, Table 2). These values are useful not only for the design phase, but also for verifying that automated visualizations are generated correctly.
- *What is the range of typical values?* Are values clustered tightly around the mean or is there a broad range of outliers? Some distributions will have a long tail that must be accounted for in visualization design. Using minutes per week of moderate physical activity as an example, a general population sample is likely to include some zero values and a mean in the tens or low hundreds. However, the outliers will have values into the thousands; a mail carrier who walks six hours per day for her/his route would report 1,800 minutes of moderate physical activity from work alone. The effect of outlier values on a visualization, such as a bar graph, can be dramatic and possibly undesirable from an aesthetic perspective (see Figure 1a). Disregarding outliers beyond an arbitrary maximum may be defensible depending upon the purpose and nature of the visualization. If not, creative solutions must be found.
- *How much rounding is optimal?* Data collected as whole numbers may best be presented in smaller units if comparison with similar values is to occur. For example, in WICER, community members expressed a preference for seeing servings of fruits or vegetables presented visually on a per-day rather than per-week basis. Therefore, although respondents typically reported servings in whole numbers, data for fruit and vegetable servings were reported back in tenths to facilitate comparison between the target individual (e.g., Maria, 2.0 servings) and the average for their reference group (e.g., other women Maria’s age, 1.8 servings). Other values, such as calories consumed per day, may best be presented in increments of five, ten, or greater. Optimal rounding should be a function of the nature of the variable and the purpose of the visualization. For instance, more generous rounding may be applied if the purpose is to support the general sense of a concept (gist comprehension) than if the purpose is to support understanding of an exact value (verbatim comprehension). If data are presented in graphs, the rounding of the axis labels requires separate consideration from that of the data points. In most cases, axis labels will look best if they are rounded to one or two increments larger than the data presented as in Figure 1a (i.e., data in tenths, axis labels in tens but selected in increments of 30).
- *Is binning appropriate?* Converting continuous values into categories (binning) may be appropriate if precise values are not essential to the message being communicated. Binning is commonly used for reporting age groups and income brackets. Examples relevant to health include Body Mass Index (BMI) categories, blood pressure categories, and the results of screening tests (e.g., positive/negative).

Note that some variables that appear continuous are functionally categorical. Short-form PROMIS measures administered outside of a computer adaptive testing environment are one such example because scoring tables are used to convert a discrete number of raw summed scores into T-scores. An 8-item short form with raw scores ranging from 8 to 40 yields 33 possible T-score values; the 4-item instrument yields only 17. Planning for and testing visualizations with only a few dozen possible T-score values is simpler than if the T-scores were actually continuous [24].

### How Are Values Interpreted?

**Figure 2:**
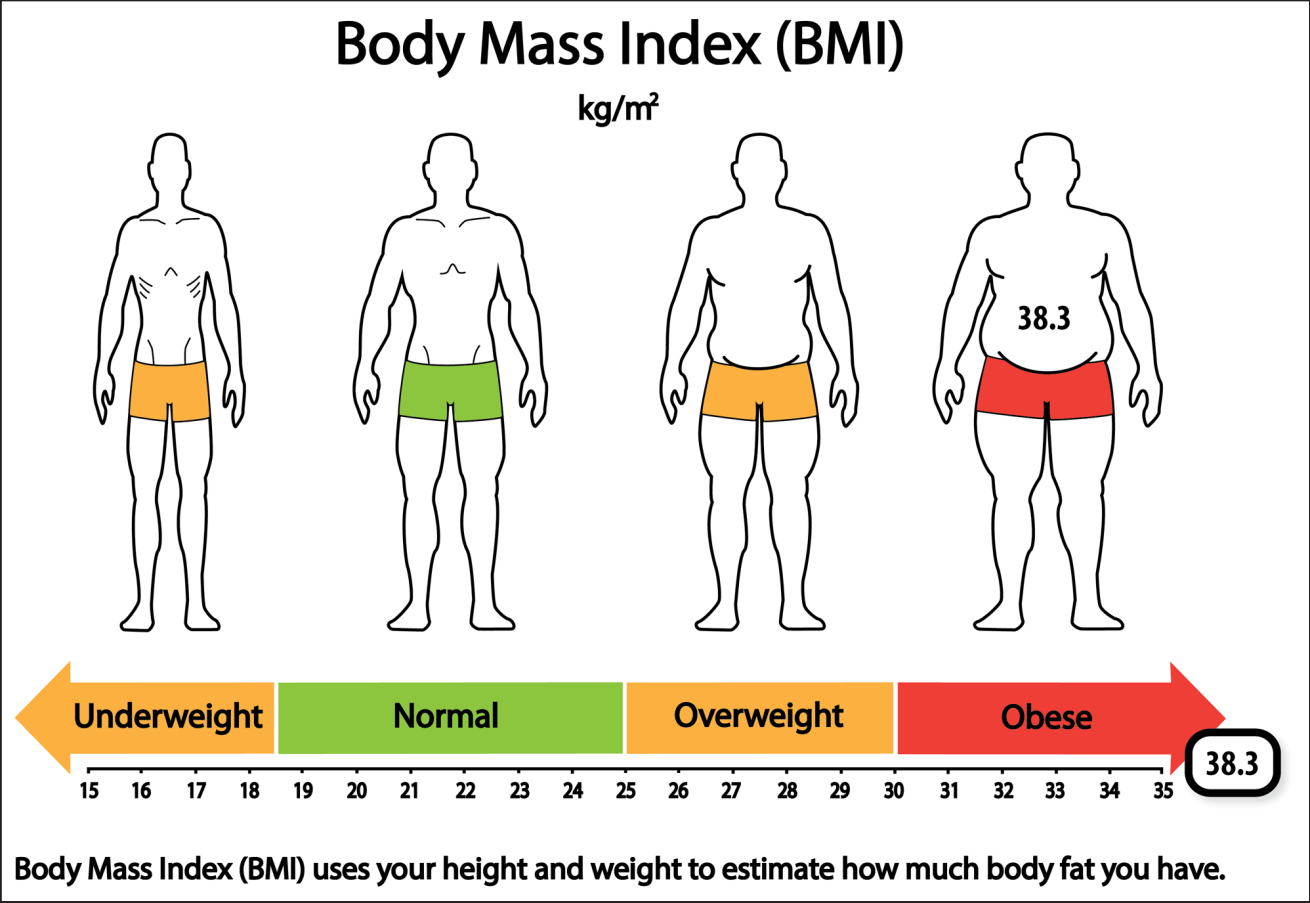
Body Mass Index (BMI) infographic from WICER showing an out-of-range value (38.3). Initially, we presented the body silhouettes (top portion) and the reference range number line (bottom portion) in participatory design sessions as separate graphical formats. We combined them into a single infographic at the suggestion of design session participants. The attributes of the data drove numerous design decisions.

Our final step in getting to know the data was understanding how values are interpreted. Considerations for value interpretation included:

- *If the variable is latent, what direction is the scoring?* Do high or low scores indicate high levels of the latent trait?
- *Are there value judgments associated with the values?* What values are considered “good” or desirable? For example, low values are preferable on the Kessler Psychological Distress Inventory; middling values are desirable for blood pressure (neither hypo- nor hypertensive); and high values on the SF1 (Item A in Table 2) indicate better overall health. The relative desirability of values can be encoded using visual cues such as color (e.g., green = healthy, red = unhealthy), visual prominence, or symbols (e.g., check marks, happy/sad faces).
- *Are there cutpoints associated with the variable?* Are there values that separate scores into meaningful categories? For example, 120 mmHg is the cutpoint between normal systolic blood pressure and pre-hypertension; 140 separates pre-hypertension from hypertension [23]. Cut points may be set by clinical practice guidelines, by instrument developers, based off of national/international norms, or even by arbitrary convention. In the event that more than one set of cutpoints exist, one might have to determine which are the most relevant for the target population and visualization purpose. When cutpoints are not equidistant as with BMI categories (e.g., normal weight spans 6.4 units whereas overweight spans 4.9 units), the designer must decide if it is more important to maintain equal-appearing intervals between the units (see Figure 2), or if aesthetics require categories to be equally sized at the expense of equality of intervals.
- *Are normed scores available for the variable?* Comparison of scores to population norms can support the interpretation of values, especially when cutpoints are not in use. For example, the Perceived Stress Scale (PSS) is a 10-item scale scored from 0 to 40 for which higher scores indicate higher levels of stress. Cutpoints are not provided by the scale developers, but they do provide a norm table showing the means (range 11.9 to 14.7) and standard deviations (range 5.0 to 7.2) for subgroups (gender, age, and race/ethnicity) of 2,387 U.S. respondents [25].

## Findings

Getting to know the data was an important early step in our visualization design process for three main reasons. One, the data attributes imposed the limitations within which the designs could occur. It is tempting to assume or wish for particular data attributes. In fact, research on designers’ processes revealed that they tended to begin designing prior to fully understanding the data and that the “designers’ inferences about data behavior were often inaccurate.”[16]. Getting to know the data well can help avoid this pitfall. Two, the precise variable meaning and the data attributes sometimes suggested design opportunities or sparked new ideas. Three, understanding the full range of values the design must accommodate prepared for inclusion of all relevant use cases and paved the way for visualization automation. The biggest design and automation challenges usually were related to handling inconvenient outlier and non-missing zero values. Figures 1a–b and 2 are presented here as case studies to illustrate these three reasons and to demonstrate how some of the considerations outlined above influenced design decisions.

Based on community feedback, we selected bar graphs as the most effective means of presenting data about physical activity (see Figure 1a and b) [10]. Given a very straightforward topic, an accurate title was all we needed to communicate the meaning of the variable. Salient data attributes for this variable were that mean values for the comparison group (i.e. “other women your age”) would fall below the recommended minimum, as would the value for the majority of the index respondents (i.e. “You”). Therefore, showing the recommended minimum as a dotted line that (in most cases) would hover above both bars, communicated the sense of a goal to be reached. The zero and outlier values were the elements that presented a challenge. In the case of non-missing zero values, we saw that the complete absence of a bar would be potentially confusing, even if the space were labeled with a zero. Our solution was to use a very shallow bar to indicate where the bar would be located, had the value been greater than zero (Figure 1a). The presence of a large outlier value (as in Figure 1b) has the effect of distorting the relative proportions of the elements within the image and the value of the recommended minimum is no longer shown on the y-axis. Although the effect of the outlier value is less than optimal from an aesthetic perspective, we opted not to attempt accommodative changes to the design for two reasons. One, the proportion of respondents possessing high outlier values was very small. Two, the primary purpose of the visualization (i.e. gist comprehension of one’s activity as compared to that of peers and the recommendation) was not compromised. It is clear that the index respondent’s physical activity far exceeds both the recommended amount and that of their peers; the precise amount of difference is not important. We could have attempted solutions such as increasing the length of the y-axis in proportion to the rest of the image so as to have less crowding around the minimum recommended value. However, the added complexity of programming for this solution was not warranted given the reasons described.

Different considerations came into play when designing an infographic to communicate BMI (see Figure 2). The BMI metric, kg/m^2^, is not an intuitive one for the general public and as such, the infographic had to support not only identification of the value in comparison to reference ranges, but also interpretation of the metric. Our research on existing BMI and body weight visualizations revealed that body silhouettes were sometimes employed to convey meaning. For that reason we elected to combine body silhouettes that evoke the meaning of the BMI categories with a number line that demarcates the reference ranges. Unlike the physical activity bar graph for which gist comprehension is sufficient, the BMI infographic had to support verbatim comprehension because one of the cutpoints is between whole values (18.5) and because personal change along the continuum is often seen in very small increments (tenths). As mentioned before, the cutpoints are not equidistant, which is a drawback aesthetically. Given the purpose of the visualization, we determined that it was more important to privilege the equality of intervals along the number line over making the BMI categories visually symmetrical. The selection of the range of values to be shown on the number line was driven by consideration of a) the values that would allow for presentation of the body silhouettes that is as visually balanced as possible, b) placement of the most common values in the center of the image, and c) adequate spacing between values for ease of reading. The drawback to the range of values selected (15–35) is that a substantial proportion of respondents have values above the range. Our solution in those cases was to place the indicator box off the number line but overlapping with the end of the arrow to suggest its placement, were the number line to be extended. We used colors (green, orange, red) to encode the value judgments associated with the categories.

## Major Themes

One major theme that emerged from our experience was the need for a systematic method to explore data attributes. For us, using such a method has made a potentially complicated and lengthy process easily manageable and more efficient, even when working with multiple variables. Our design process during NHiRP was dramatically shorter than it was for WICER. Some of the efficiency gains can be attributed to the numerous other lessons learned unrelated to understanding the attributes of the data. However, having implemented our method, we prepared far fewer initial prototypes overall and required fewer refinement iterations, thus reducing time and cognitive load. Also, we did not experience the frustration of having to discard prototypes because of mismatch with the data attributes as we had during WICER. We anticipate that incorporating the data attributes into a Style Guide (a tool to communicate design specifications to the programmer [11]) will reduce the communication burden and workload when it comes time to program EnTICE [3] to tailor the infographics. Any research methods designed to measure the aforementioned benefits could be used to quantify the value of our method.

**Figure 3:**
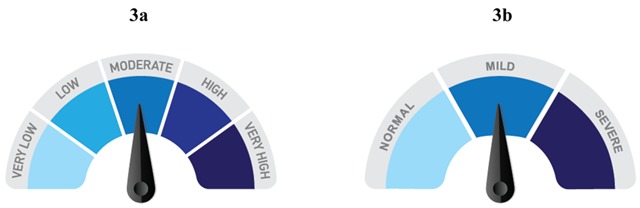
At left **(3a)** is a visual element used in a WICER infographic to display scores for the PHQ-9. At right **(3b)** is an early NHiRP prototype to show a score on the Geriatric Depression Scale (GDS) using the same concept. The loss of categories because of a difference in instrument scoring meant an unacceptable loss of visual interest and the decision to pursue other design options. The final GDS infographic is a three-category reference range number line similar to the one in Figure 2, but with a blue gradient instead of distinct color blocks.

The second major theme has been that data attributes are like two sides of the same coin; the limitations of the data can narrow design options but can also prompt creative solutions and innovative design opportunities. The process of understanding the precise meaning of a variable (e.g., meeting a diagnostic threshold for depression vs. intensity of symptoms) is probably where design opportunities are most likely to present themselves. However, even awareness of the specific values and cutpoints associated with a variable can inform decisions about symmetry and visual balance, or about the number of colors that will be required in a palette (see Figure 3a and b). Existing tools such as the Data Viz Project (http://www.datavizproject.com) and Visualizing Health (http://www.vizhealth.org/) have filtering capabilities to aid in the selection of an appropriate graphical format. Given a deeper understanding of how data attributes influence selection of optimal format, palette, symmetry, etc., tools such as these could be refined. For example, filtering for a format appropriate for an item with bipolar response options and a neutral midpoint (Table 2, Item B) could return a symmetrical format with an odd number of categories, whereas a similar item without a neutral midpoint (Table 2, Item C) would return a format with an even number of categories.

### Limitations

One limitation to our method is that it assumes access to summary statistics, which not everyone may have. In such instances, most steps of the method may be completed but users will be limited in their ability to identify the values most likely to occur in the data, and in some cases, will not be able to specify minimum and/or maximum values. Another limitation is that we have applied our method to a few dozen variables, at most. Other variable types may necessitate asking additional questions to the ones presented here. For instance, further innovation may be needed for adequate exploration of datasets that employ repeated measures or in which multivariate relationships are important. We invite teams engaged in similar work to share their own discoveries with respect to the process of exploring data attributes, particularly when the data are complex.

## Conclusion

In this paper we have presented a systematic method to understanding the attributes of patient reported outcomes data in preparation for automated visualization. The value of the method is that it contributes to an efficient design process, uncovers design opportunities, and alerts the designer to design challenges. A streamlined visualization development process is important given the increasing utility of visualization as a health communication tool. This is increasingly significant as a component of patient engagement whether in the care context as part of efforts to integrate PROs into electronic health records or to promote self-management or in the research context as part of research results reporting and sustaining longitudinal cohorts.
